# Antitumor Efficacy of Arylquin 1 through Dose-Dependent Cytotoxicity, Apoptosis Induction, and Synergy with Radiotherapy in Glioblastoma Models

**DOI:** 10.3390/biomedicines12040907

**Published:** 2024-04-19

**Authors:** Ann-Shung Lieu, Yu-Chi Pan, Jia-Hau Lee, Yuan-Chin Hsieh, Chien-Ju Lin, Ya-Ling Hsu, Kung-Chao Chang, Shih-Hsun Kuo, Tzu-Ting Tseng, Hung-Pei Tsai

**Affiliations:** 1 Division of Neurosurgery, Department of Surgery, Kaohsiung Medical University Hospital, Kaohsiung 80708, Taiwan; e791125@gmail.com (A.-S.L.); cawaii7992@gmail.com (T.-T.T.); 2 Department of Surgery, School of Medicine, College of Medicine, Kaohsiung Medical University, Kaohsiung 80756, Taiwan; 3 Graduate Institute of Medicine, College of Medicine, Kaohsiung Medical University, Kaohsiung 80756, Taiwan; rita10732@gmail.com (Y.-C.P.); yainghsu@kmu.edu.tw (Y.-L.H.); 4 National Institute of Cancer Research, National Health Research Institutes, Tainan 70456, Taiwan; 120307@nhri.edu.tw; 5 School of Medicine for International Students, I-Shou University, Kaoshiung 82445, Taiwan; hyc1014@isu.edu.tw; 6 School of Pharmacy, College of Pharmacy, Kaohsiung Medical University, Kaohsiung 80756, Taiwan; mistylin@kmu.edu.tw; 7 Drug Development and Value Creation Research Center, Kaohsiung Medical University, Kaohsiung 80756, Taiwan; 8 Department of Pathology, National Cheng Kung University Hospital, College of Medicine, National Cheng Kung University, Tainan 70101, Taiwan; changkc@mail.ncku.edu.tw; 9 Department of Radiation Oncology, Kaohsiung Medical University Hospital, Kaohsiung 80708, Taiwan; 1020001@kmuh.org.tw

**Keywords:** Arylquin 1, glioblastoma, antitumor efficacy, cytotoxicity, apoptosis, radiotherapy

## Abstract

Glioblastoma (GBM), the most aggressive form of brain cancer, is characterized by rapid growth and resistance to conventional therapies. Current treatments offer limited effectiveness, leading to poor survival rates and the need for novel therapeutic strategies. Arylquin 1 has emerged as a potential therapeutic candidate because of its unique mechanism of inducing apoptosis in cancer cells without affecting normal cells. This study investigated the efficacy of Arylquin 1 against GBM using the GBM8401 and A172 cells by assessing its dose-dependent cytotoxicity, apoptosis induction, and synergy with radiotherapy. In vitro assays demonstrated a significant reduction in cell viability and increased apoptosis, particularly at high concentrations of Arylquin 1. Migration and invasion analyses revealed notable inhibition of cellular motility. In vivo experiments on NU/NU nude mice with intracranially implanted GBM cells revealed that Arylquin 1 substantially reduced tumor growth, an effect magnified by concurrent radiotherapy. These findings indicate that by promoting apoptosis and enhancing radiosensitivity, Arylquin 1 is a potent therapeutic option for GBM treatment.

## 1. Introduction

Glioblastoma (GBM) is the most common and aggressive primary brain tumor in adults, with an incidence rate of 6.13 per 100,000 individuals [1]. The risk factors for this tumor vary and are difficult to pinpoint, and its clinical progression is often lethal. Diagnosis primarily relies on histopathological results, but assessments of molecular markers, such as isocitrate dehydrogenase (IDH) mutations and O6-methylguanine DNA methyltransferase (MGMT) promoter methylation, along with more extensive molecular analyses, are increasingly being utilized for prognostic subtyping and tailored treatment strategies. Standard treatments for GBM include traditional surgery, radiotherapy (RT), and alkylating agent chemotherapy [2]; however, the prognosis remains poor. Advanced age, poor physical condition, and incomplete surgical resection are recognized as adverse prognostic factors [3,4]. Although short-term survival rates have gradually improved over time, the five-year survival rate remains relatively stable at only 5.8% after post-diagnosis [5,6,7].

Arylquin 1 has been identified as a secretagogue of prostate apoptosis response-4 (Par-4) in normal cells. This compound binds to vimentin and facilitates the release of Par-4, which is then secreted [8,9]. Par-4 was originally identified by the differential screening of genes that were upregulated following the induction of programmed cell death in prostate cancer cells [10]. Most studies on Par-4 have focused on its apoptotic effects mediated by intracellular Par-4. The apoptotic effects of Par-4 involve activation of the Fas death receptor signaling pathway and inhibition of cellular survival mechanisms [11]. Recent research has shown that normal cells secrete the Par-4 protein, and extracellular Par-4 induces apoptosis in cancer cells specifically through interaction with the 78 kDa glucose-regulated protein (GRP78) on the cell surface [12]. For example, in the Eµ-TCL1 leukemia model, increased Par-4 expression impedes leukemogenesis predominantly by downregulating NF-κB signaling [13]. Furthermore, Par-4 plays a critical role in preventing the invasion and metastasis of cancer cells. It has been established that inducing intracellular Par-4 can halt epithelial–mesenchymal transition (EMT) and invasion in prostate and breast cancer cells [14]. Moreover, the extracellular or secreted forms of Par-4 also contribute to its anti-cancer properties. These forms are capable of blocking MMP-2-mediated invasion and angiogenesis, observed in human cervical and prostate cancer cells [14,15]. Arylquin 1, a Par-4 secretagogue, induces non-apoptotic cell death associated with lysosomal membrane permeabilization (LMP) in various cancer cells [16,17,18]. This study investigated the effect of Arylquin 1 on GBM and examined its potential as a novel therapeutic agent against this aggressive form of brain cancer.

## 2. Materials and Methods

### 2.1. Cell Lines and Cell Culture

GBM8401 and A172 were the GBM cell lines. GBM8401 was obtained from the Bioresource Collection and Research Center (BCRC, Taipei, Taiwan; 60163), and A172 was obtained from the American Type Culture Collection (ATCC, Manassas, VG, USA; CRL-1620). GBM8401 cells were cultured in Roswell Park Memorial Institute (RPMI) medium supplemented with 10% fetal bovine serum (FBS). A172 cells were cultured in DMEM supplemented with 10% FBS. All cells were incubated at 37 °C with 5% CO_2_.

### 2.2. Cell Viability Assay

The GBM8401 and A172 cells were prepared by resuspending the cells in a culture medium containing 10% FBS. The suspension was placed in 24-well plates with each well containing approximately 30,000 cells in a total volume of 0.5 mL. These cells were cultured in an environment with 5% CO_2_, full humidity, and a temperature of 37 °C for 72 h. Post-incubation, the cell viability was assessed using the trypan blue exclusion method after exposing the cells to various concentrations of Arylquin 1, ranging from 0 to 10 μM in a graded manner (0, 0.5, 1, 1.75, 2.5, 3.75, 5, and 10 μM).

### 2.3. Cell Migration Assay

Cell migration was evaluated using a wound healing assay kit (ibidi, Gräfelfing, Germany; 80209) designed for 24-well plates. The assay was initiated by coating Matrigel (Sigma, Saint Louis, MO, USA, E1270) in the plates and incubating them at 37 °C for 12 h. Cells were seeded at a concentration of 1 × 10^6^ cells/mL, using 70 μL per well. Cells were then left to adhere and grow for 24 h. After this period, the cells were gently washed twice with phosphate-buffered saline (PBS) to remove non-adherent cells. The migration process was then initiated by treating the cells with 0.5 μM Arylquin 1, and the cell movement towards the wound area was documented by taking photographs.

### 2.4. Invasion Assay

Invasion assays were conducted in vitro using a Transwell assay kit. Initially, cells were seeded at 10,000 cells per insert. The lower chamber of the Transwell setup was filled with 0.5 mL of medium containing 0.5 μM Arylquin 1 to act as a chemoattractant. The setup was incubated for 24 h to allow cell invasion through the membrane. After incubation, the cells that did not migrate through the membrane and remained on the upper surface were gently removed using a cotton swab. Cells that successfully invaded the lower surface of the insert through the membrane were fixed with methanol, stained with crystal violet for visualization, and photographed. The invasion was quantified by counting the number of cells in six randomly selected high-power microscopic fields.

### 2.5. Colony Formation

GBM8410 and A172 cells were cultured in six-well plates with varying densities (100, 200, 400, 1000, and 10,000 cells per well) and were then treated with Arylquin 1 at concentrations of 0, 1, 2.5, and 5 µM. Following this treatment, the cells were subjected to radiation at doses of 0, 1, 2, 4, and 8 Gy using a linear accelerator, with the procedure carried out at ambient temperature. After 10 days of incubation, the cells were stained with a 0.5% crystal violet solution to facilitate colony counting. Colony formation was quantified by adjusting the plating efficiency (PE) to yield the surviving fraction (SF) in comparison with untreated controls. Plating efficiency was determined as the ratio of colonies observed to the initial number of cells plated, multiplied by 100. The surviving fraction was calculated as the quotient of the number of colonies formed by the product of the number of cells initially plated and the plating efficiency, divided by 100 (%).

### 2.6. Flow Cytometry

The GBM8401 and A172 cells were plated in six-well plates, with each well containing 500,000 cells. The cells were cultivated for 72 h to reach logarithmic phase growth. Following this, the cells were treated with varying concentrations of Arylquin 1 (0, 1, 2.5, and 5 µM) in complete culture medium for another 72 h. Subsequently, 1 million cells from each treatment group were isolated and resuspended in 100 µL of Annexin V and dead cell reagent (Merck Millipore, Warsaw, Poland) for 20 min at ambient temperature, using the medium as a control group. The apoptosis levels were quantified using a Muse^®^ Cell Analyzer (Merck Millipore), employing fluorescence detection at yellow–red wavelengths (576 to 680 nm) with excitation at 532 nm. Separately, for the cell cycle analysis, cells were seeded at a density of 10,000 cells per well and incubated at 37 °C in a medium enriched with 10% FBS. Post-incubation with Arylquin 1 (concentrations of 0, 1, 2.5, and 5 µM) for 72 h, the cells underwent washing with PBS and centrifugation. The pellet was then fixed in cold 70% ethanol and stored at 20 °C pending further analysis. Prior to cell cycle analysis, the cells were washed again with PBS and prepared according to the guidelines provided by the cell cycle assay manufacturer.

### 2.7. Animal Models

All immunodeficient and NU/NU mice were obtained from the LASCO Laboratory Animal Center (Taipei, Taiwan). The experimental procedures involving these mice were approved by the Institutional Animal Care and Use Committee of Kaohsiung Medical University (IACUC Approval No: 109252) and strictly adhered to the established guidelines for the care and use of laboratory animals. The mice were maintained in an environment with a constant temperature of 24 °C and subjected to normal light/dark cycles (6:00 a.m. to 6:00 p.m.) with unlimited access to standard feed. For the study, each mouse received an intracranial injection of GBM8401 cells tagged with luciferase (100,000 cells in 5 μL) into their striatum. 1 week post-injection, an intraperitoneal injection of 1 or 5 µM/kg Arylquin 1 was administered to the mice. In the radiosensitivity tests, 1 week post-injection, the mice underwent biweekly 2 Gy radiation treatments. Tumor progression and development were monitored using in vivo bioluminescence imaging. This imaging was conducted with the Xenogen IVIS^®^ Spectrum Noninvasive Quantitative Molecular Imaging System (J&H, Hongkong, China; IVIS Lumina LT 2D) at intervals of 7, 14, and 21 days after the GBM cells were administered.

### 2.8. Data Analysis

Statistical analyses were conducted using the SPSS software (version 19.0; Chicago, IL, USA). Western blot results were evaluated using Lane 1D^TM^ software. We employed one-way ANOVA to ascertain differences in proliferation, migration, and invasion assay outcomes. Results yielding a *p*-value of less than 0.05 were deemed statistically significant.

## 3. Results

### 3.1. Dose-Dependent Cytotoxicity of Arylquin 1 in GBM8401 and A172 Cells

**Figure 1:**
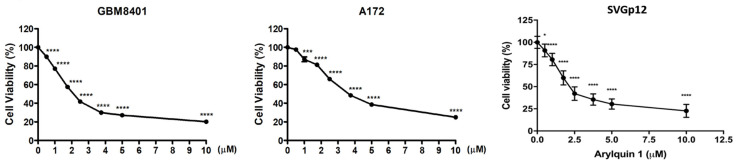
Effect of Arylquin 1 on glioblastoma (GBM) cell viability. The graph illustrates the dose-dependent cytotoxicity of Arylquin 1 on GBM8401 and A172 cells. Cell viability was assessed at various concentrations of Arylquin 1 (ranging from 0 to 10 μM). A steep decline in cell viability was observed with increasing concentrations of Arylquin 1, with significant cytotoxic effects noticeable at lower concentrations that became more pronounced with each successive increase in dosage. Statistical significance is indicated by asterisks, with one asterisk (*) denoting *p* < 0.05, three asterisks (***) denoting *p* < 0.01, four asterisks (****) denoting *p* < 0.0001, reflecting a highly significant reduction in cell viability after Arylquin 1 treatment.

To demonstrate the therapeutic effect of Arylquin 1 in GBM cells, MTT assay detected cell viability in 0, 0.5, 1, 1.75, 2.5, 3.75, 5, and 10 μM in GBM8401 and A172 cells. In GBM8401 cells, the influence of Arylquin 1 on cell viability displayed a clear dose-response relationship. While the initial concentration of 0.5 µM does not significantly alter viability compared to untreated cells, subsequent concentrations from 1 µM Arylquin 1 onwards show a statistically significant reduction in cell survival, suggesting a substantial effect on cell viability (*p* < 0.0001) (Figure 1). As the concentration of Arylquin 1 increases, cell viability decreases, with the most pronounced effect observed at 10 µM (*p* < 0.0001) (Figure 1). The half-maximal inhibitory concentration (IC50) was established at 2.12 µM, which highlights the drug’s efficacy in reducing GBM8401 cell viability in a concentration-dependent manner. In A172 cells, the response to Arylquin 1 differed. There was no significant change in cell viability at the lower concentration of 0.5 µM, similar to GBM8401 cells. However, in subsequent concentrations from 1 µM, the effect became statistically significant, indicating the beginning of a considerable response to Arylquin 1 (*p* < 0.0001) (Figure 1). As the dose increased, cell viability continued to decline, reflecting consistent dose-dependent sensitivity to the drug. The IC50 for A172 cells was calculated at 3.7 µM, which is slightly higher than that of GBM8401 cells, suggesting a differential sensitivity to the treatment. The highest concentration of 10 µM exceptionally decreased cell viability (*p* < 0.0001), confirming the robust inhibitory effect of Arylquin 1 on A172 cell viability (Figure 1). These findings indicated that GBM8401 and A172 cells exhibited an Arylquin 1 dose-dependent decrease in viability. In this study, SVGp12 cells are used as a representative normal glial cell line for comparison. Though the study primarily focuses on the cytotoxic effects of Arylquin 1 on GBM cell lines, Arylquin 1 also demonstrates toxicity towards SVGp12 cells (Figure 1). This suggests that although Arylquin 1 is effective in decreasing the viability of cancerous glial cells, it also affects non-cancerous glial cells.

### 3.2. Induction of Apoptosis and Cell Cycle Arrest by Arylquin 1 in GBM8401 and A172 Cells

**Figure 2:**
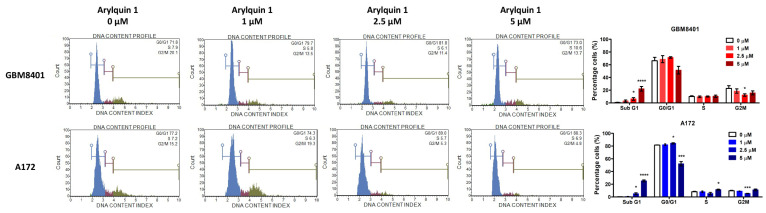
Arylquin 1 modulates cell cycle distribution in GBM8401 and A172 cells. Flow cytometry histograms display the DNA content profiles, reflecting the distribution of cells across different phases of the cell cycle in GBM8401 and A172 cells treated with Arylquin 1 at concentrations of 0, 1, 2.5, and 5 μM. Histograms represent the percentage of cells in the G0/G1, S, and G2/M phases, with the Sub G1 peak indicating apoptotic cells. The bar graphs quantify the percentage of cells in each phase of the cell cycle corresponding to each treatment. GBM8401 cells showed an increase in the Sub G1 population with higher doses of Arylquin 1, indicating increased apoptosis. Similarly, A172 cells exhibited a dose-dependent increase in Sub G1, with a marked escalation at 2.5 μM and 5 μM, suggesting enhanced apoptosis. Notable changes in cell cycle phases, such as a decrease in the S and G2/M phases at higher concentrations, indicated cell cycle arrest and reduced proliferation. Asterisks indicate statistical significance compared to the untreated control: * *p* < 0.05, *** *p* < 0.001 and **** *p* < 0.0001.

**Figure 3:**
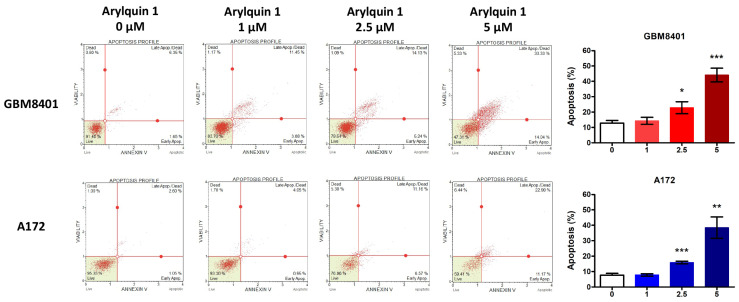
Arylquin 1 induces dose-dependent apoptosis in GBM8401 and A172 cells. Flow cytometric apoptosis profiles of GBM8401 and A172 cells treated with various concentrations of Arylquin 1 (0, 1, 2.5, and 5 μM). The quadrants represent live (bottom left), early apoptotic (bottom right), late apoptotic/dead (top right), and dead (top left) cells. The corresponding bar graphs summarize the percentage of apoptotic cells, combining the early and late apoptotic populations, for each treatment group. Data indicate a significant increase in apoptosis with higher concentrations of Arylquin 1 in both cells, with GBM8401 showing a notable rise in apoptosis at both at 2.5 μM (* *p* < 0.05) and 5 μM (*** *p* < 0.001) and A172 cells displaying a significant increase at both 2.5 μM (** *p* < 0.01) and 5 μM (*** *p* < 0.001) compared to the untreated control.

To demonstrate the mechanism of Arylquin 1 in GBM cells, flow cytometry assessed the cell cycle with different doses (1, 2.5, and 5 μM) of Arylquin 1 in GBM8401 and A172 cells. Following the cell cycle assay, the mechanism of action of Arylquin 1 in GBM cells was elucidated through flow cytometry, assessing apoptosis and cell cycle progression at various concentrations (1, 2.5, and 5 μM) of Arylquin 1 in both GBM8401 and A172 cells. In GBM8401 cells, the sub-G1 population, indicative of apoptotic cells, exhibited a significant increase with higher concentrations of Arylquin 1, particularly at 5 μM, where there was a rise to 22.2%, compared to the untreated control at 0.8% (Figure 2). This suggested an increase in the rate of apoptosis. Concurrently, there was a discernible shift in cell cycle phases. The proportion of cells in the G0/G1 phase was augmented at 1 and 2.5 μM of Arylquin 1 but saw a decrease at 5 μM, which implies a cell cycle arrest at lower doses and potential cell death at higher doses (Figure 2). The S phase remained relatively stable under all treatment conditions, suggesting that the primary effect of Arylquin 1 was not on DNA synthesis (Figure 2). In contrast, the number of cells in the G2/M phase decreased with increasing Arylquin 1 concentration, indicating a disruption in cell cycle progression, possibly in the mitotic phase. In A172 cells, a dose-dependent increase in the sub-G1 cell population was also observed, with a stark increase to 25.25% at 5 μM (from a baseline of 0.4%), indicating pronounced apoptosis (Figure 2). The G0/G1 phase showed an increase in cells at 1 and 2.5 μM, but a substantial reduction at 5 μM, aligning with the results seen in the GBM8401 cells, suggesting a similar pattern of cell cycle arrest followed by apoptosis at the highest concentration. The S phase population decreased with higher doses, notably at 2.5 μM, reflecting a possible inhibition of DNA synthesis or progression through the S phase. The G2/M phase population decreased at 2.5 μM but increased slightly at 5 μM, which could indicate cell cycle perturbations specific to the A172 cell type at this concentration (Figure 2). To confirm the results from the cell cycle assay, flow cytometry detected the apoptosis with different doses (1, 2.5, and 5 μM) of Arylquin 1 in GBM8401 and A172 cells. Investigation of the apoptotic effects of Arylquin 1 on GBM cells yielded compelling results, as evidenced by the data for GBM8401 and A172 cells. In the absence of Arylquin 1, GBM8401 cells exhibited a baseline apoptosis rate of 12.894%, which increased incrementally with the administration of the drug, peaking at 44.134% upon treatment with 5 μM Arylquin 1 (Figure 3). This denotes a more than three-fold increase in the apoptotic rate at the highest concentration tested compared to the control. In a similar pattern, A172 cells showed an initial apoptosis rate of 7.746% at 0 μM, which also escalated with rising concentrations of Arylquin 1, culminating in a substantial increase to 38.4825% at 5 μM (Figure 3). The bar graphs illustrate these increases in apoptosis rates with statistical markers that signify the level of significance compared to the control group (0 μM). In GBM8401 cells, there is a statistically significant rise in apoptosis at 2.5 μM (* *p* < 0.05) and an even more significant effect at 5 μM (*** *p* < 0.001). The A172 cells demonstrate a statistically significant response starting from 2.5 μM (** *p* < 0.01) and, akin to GBM8401, a very significant increase at 5 μM (*** *p* < 0.001). These findings indicated a clear dose-dependent increase in apoptosis when GBM8401 and A172 cells were treated with Arylquin 1 (Figure 3).

### 3.3. Inhibitory Effect of Arylquin 1 on Invasion and Migration of GBM8401 and A172 Cells

**Figure 4:**
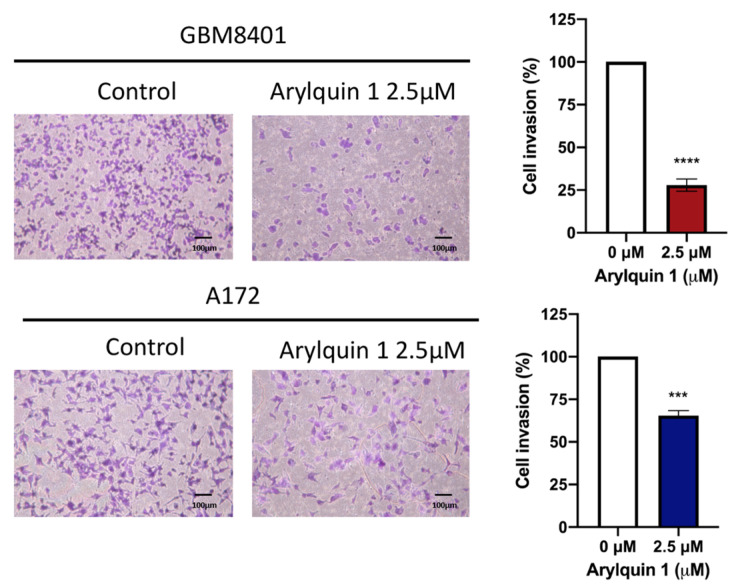
Effects of Arylquin 1 on GBM cell invasiveness in Matrigel invasion assays. Microscopic images depicting the invasive properties of GBM8401 and A172 cells through Matrigel matrices following treatment with Arylquin 1. The left panels show untreated control cells with dense cellular invasion, whereas the right panels show cells post–Arylquin 1 treatment, displaying reduced invasion. The quantified invasion rates for GBM8401 and A172 cells illustrated a significant decline in cell invasion upon Arylquin 1 treatment compared to the controls. Asterisks indicate statistical significance compared to the untreated control: *** *p* < 0.001 and **** *p* < 0.0001.

**Figure 5:**
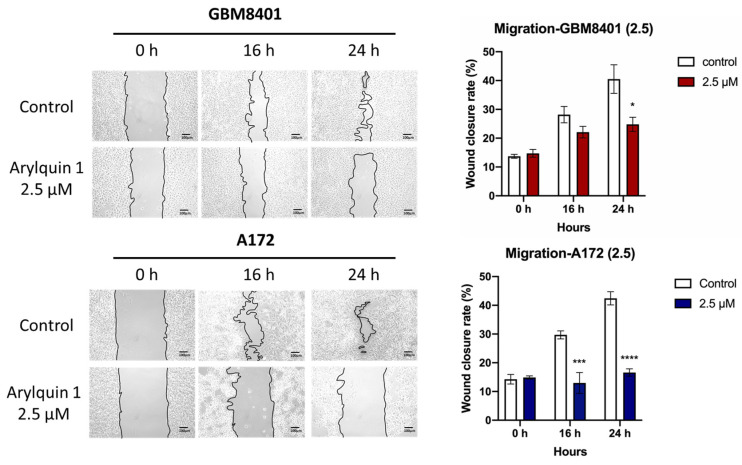
Impact of Arylquin 1 on GBM cell migration in a wound healing essay. Displayed are the results of a wound healing assay performed on GBM8401 and A172 cells to assess the impact of Arylquin 1 on cellular migration. Time-lapse images captured at 0, 8, 12, and 24 h post–Arylquin 1 application documented the changes in wound closure. The images show a noticeable decrease in the wound closure rate of GBM8401 cells after Arylquin 1 treatment compared to that of the control, which was more pronounced at higher concentrations of the drug. A172 cells exhibited a similar reduction in wound-healing capability after treatment. The bar graphs on the right quantitatively depict the percentage of wound closure over time, underlining the inhibitory effect of Arylquin 1 on cell migration in both GBM8401 and A172 cells, with a notable dose-dependent reduction in the migration rate relative to the untreated controls. Asterisks indicate statistical significance compared to the untreated control: * *p* < 0.05, *** *p* < 0.001 and **** *p* < 0.0001.

The cell invasion capabilities of the GBM8401 and A172 cells were assessed to understand the impact of Arylquin 1 treatment on GBM cell invasiveness using a Matrigel invasion assay. After treatment with Arylquin 1, there was a noticeable decline in the invasion rates of both cells compared with the untreated controls. In GBM8401 cells, the untreated control group showed robust invasive behaviour, with an average invasion rate of 71% (Figure 4). However, following the administration of Arylquin 1, a substantial decrease in invasiveness was observed as the invasion rate decreased to approximately 18%. This significant decrease implied a strong inhibitory effect of Arylquin 1 on the invasive potential of GBM8401 cells (*p* < 0.0001) (Figure 4). The A172 cells exhibited a similar trend with the control group exhibiting an average invasion rate of approximately 51% (Figure 4). After treatment with Arylquin 1, A172 cells experienced a reduction in invasion to an average rate of only 35.7 % (*p* < 0.001) (Figure 4). This marked decline indicates the potent anti-invasive properties of Arylquin 1. These findings indicate that Arylquin 1 significantly reduces the invasive capacity of GBM cells, and the marked reduction in invasion rates after treatment suggests that Arylquin 1 may interfere with key processes or pathways involved in GBM cell invasiveness. Cell migration was assessed using a wound-healing assay to gauge the effects of Arylquin 1 treatment on GBM8401 and A172 cells. The extent of wound closure was measured at 0, 16, and 24 h after administration of Arylquin 1. In GBM8401 cells, the control group demonstrated rapid wound healing, starting with an average of 13% closure at 0 h, which increased to approximately 28% at 16 h and 30% at 24 h, indicating active migration (Figure 5). However, treatment with Arylquin 1 resulted in a pronounced delay in wound closure. The migration percentages in the treated group were reduced to approximately 15% at 0 h, 22% at 16 h, and 25% at 24 h, indicating a significant impairment in the migratory ability of the cells at 24 h (*p* < 0.05) (Figure 5). For A172 cells, the control group started with a similar initial migration percentage of approximately 1.4%, which increased to approximately 14% at 0 h, 12% at 16 h, and 28% at 24 h, indicating a moderate migration rate (Figure 5). However, post-treatment with Arylquin 1, the migration rate markedly decreased to an average of 15% at 0 h, 22% at 16 h, and 25% at 24 h, indicating a substantial reduction in cell migration (*p* < 0.001 at 16 h; *p* < 0.0001 at 24 h) (Figure 5). These results suggest that Arylquin 1 substantially hampered the migratory capacity of both GBM cells, with both GBM8401 and A172 cells exhibiting significantly lower rates of wound closure than their respective controls. This reduction in cell migration underscores Arylquin 1’s potential as an inhibitor of the migratory and invasive properties of GBM cells.

### 3.4. Synergistic Effects of Arylquin 1 and Radiation on Cell Survival in GBM8401 and A172 Cells

**Figure 6:**
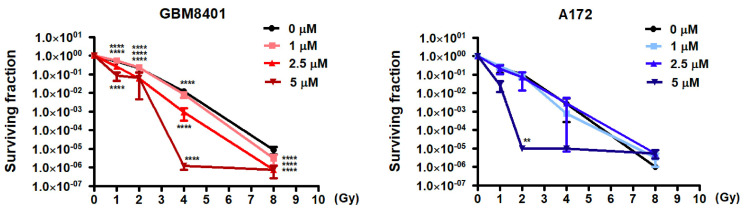
Enhancing radiosensitivity in GBM cells with Arylquin 1 treatment. The graphs display the surviving fractions of GBM8401 and A172 cells post-irradiation with increasing doses of radiation (0 to 8 Gy) in the presence of varying concentrations of Arylquin 1 (0, 1, 2.5, and 5 μM). For GBM8401 cells, survival decreased with increasing doses of Arylquin 1, indicating a synergistic effect that enhanced radiosensitivity. The surviving fraction was dramatically reduced at the highest concentration of Arylquin 1, particularly at higher radiation doses, as indicated by the steep decline in the red lines. A similar pattern was observed in A172 cells, where Arylquin 1 treatment leads to a substantial decrease in cell survival upon radiation exposure, suggesting increased radiosensitivity. These results demonstrated that Arylquin 1 could potentially be used to sensitize GBM cells to radiation therapy, lowering the surviving fraction of cells more effectively than radiation alone. Statistical significance is denoted by asterisks, indicating a potentiated reduction in survival at each dose level compared to the control. Asterisks indicate statistical significance compared to the untreated control: ** *p* < 0.01, **** *p* < 0.0001.

The standard operating procedures for treatment of GBM include chemotherapy and RT after surgery. The combined effect of Arylquin 1 and radiation on the survival of GBM8401 and A172 cells was methodically assessed, aligning with the standard treatment protocol of surgery followed by adjuvant chemotherapy and RT. For GBM8401 cells, there was a pronounced decrease in the surviving fraction as the dosages of both Arylquin 1 and radiation increased. Radiation reduced cell survival, and the presence of Arylquin 1 amplified this effect. Notably, at 5 μM Arylquin 1, the surviving fraction fell sharply to 0.086 at 1 Gy and drastically lowered to 7.5 × 10^−7^ at 8 Gy, indicating a potentiated response to RT when combined with the drug (Figure 6). Similarly, A172 cells exhibited decreased survival with increasing doses of Arylquin 1 and radiation. At the highest concentration of Arylquin 1 (5 μM), the surviving fraction at 1 Gy was notably diminished to 0.028 and approached a minimal value at higher radiation doses (Figure 6), consistent with the findings in GBM8401 cells. These results suggest that Arylquin 1 can significantly enhance the sensitivity of GBM cells to radiation, potentially offering a beneficial synergistic effect for the treatment of GBM by effectively reducing the surviving fraction of tumor cells at lower doses of radiation.

### 3.5. Synergistic Inhibition of Intracranial Tumor Growth by Arylquin 1 and Radiation Therapy in a Murine Model

**Figure 7:**
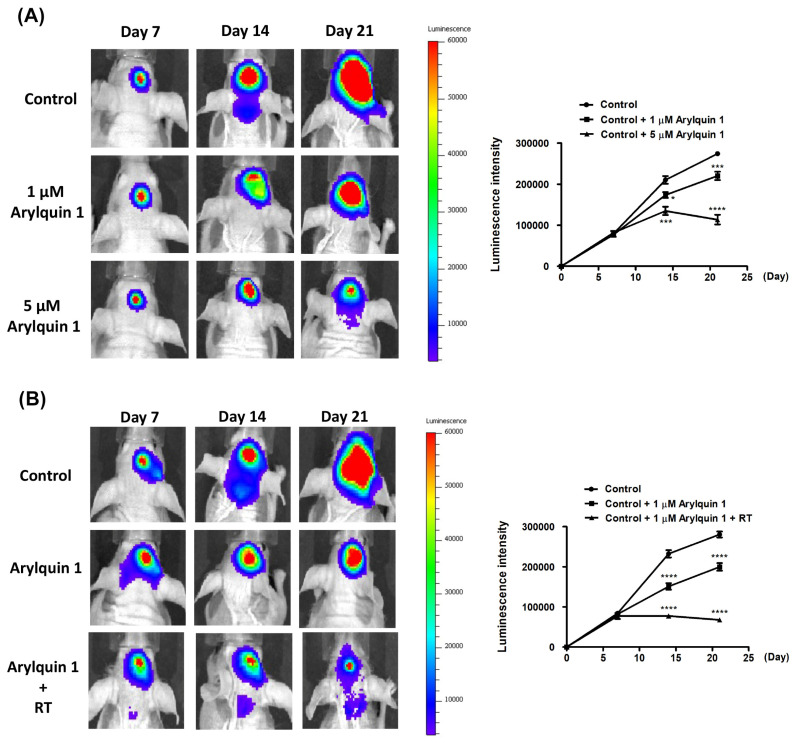
**Arylquin 1 impact on tumor growth with and without radiotherapy.** (**A**) The luminescence intensity measurements reveal tumor progression over time in groups treated with different concentrations of Arylquin 1. Compared to the control, both 1 μM and 5 μM doses of Arylquin 1 manifest a clear reduction in tumor growth, with the 5 μM concentration exhibiting a more pronounced suppressive effect. (**B**) This panel highlights the comparative tumor progression between the control group, a group treated with 1 μM Arylquin 1, and another group receiving a combination of 1 μM Arylquin 1 and radiotherapy (RT). Treatment with 1 μM Arylquin 1 shows a reduction in tumor growth, and, when combined with RT, this effect is significantly amplified, indicating a synergistic interaction that greatly diminishes tumor proliferation. Asterisks indicate statistical significance compared to the untreated control: * *p* < 0.05, *** *p* < 0.001 and **** *p* < 0.0001.

Our study assessed the impact of daily intraperitoneal injections of Arylquin 1 on the growth of intracranial tumors in a murine model. Luminescence intensity, which correlates with tumor size, was monitored on days 7, 14, and 21 after tumor implantation. The control group, which did not receive Arylquin 1, showed a marked increase in luminescence intensity, indicating rapid tumor growth over the course of three weeks. In contrast, the group treated with 1 μM Arylquin 1 displayed a more moderate increase in luminescence intensity, suggesting that the treatment was able to slow down tumor growth (*p* < 0.05 at day 14; *p* < 0.001 at day 21) (Figure 7A). The most significant effect was seen in the group treated with 5 μM Arylquin 1, which showed a substantially lower luminescence intensity at each measured time point compared to the control groups (*p* < 0.001 at day 14; *p* < 0.0001 at day 21) (Figure 7A). These results indicate that Arylquin 1, especially at a higher concentration of 5 μM, effectively suppresses tumor growth when administered daily after the initial tumor implantation. The reduced luminescence intensity on days 7, 14, and 21 post-implantation suggests that Arylquin 1 may be a potent therapeutic agent for inhibiting tumor progression. In addition, following the radiosensitivity test, luminescence intensity was used as a proxy for tumor growth in a murine model, where the impact of Arylquin 1 and RT on tumor progression was evaluated. Starting from day 7 post–tumor implantation, the mice received daily intraperitoneal injections of 1 μM Arylquin 1. In addition, the RT group underwent biweekly sessions of 2 Gy radiation. In the control group, a significant increase in luminescence intensity was observed, indicating tumor growth over the 21 days. The group treated with only 1 μM Arylquin 1 showed a noticeable decrease in the rate of luminescence intensity increase, suggesting some tumor growth inhibition (*p* < 0.0001 at day 14 and day 21) (Figure 7B). However, the most profound inhibition of tumor growth was seen in the group treated with both 1 μM Arylquin 1 and RT. This combined treatment group exhibited substantial suppression of luminescence intensity, indicating a pronounced reduction in tumor growth compared to the control group (*p* < 0.0001 at day 14 and day 21) (Figure 7B). These findings suggest that while Arylquin 1 contributes to the reduction of tumor growth, its combination with RT is significantly more effective. The reduced luminescence intensity in the combined treatment group on days 7, 14, and 21 post–tumor implantation indicated a synergistic effect between Arylquin 1 and radiation in mitigating tumor progression.

## 4. Discussion

Arylquin 1, a protein known for its tumor suppression capabilities, serves as a secretagogue of prostate apoptosis response-4 (Par-4) in healthy cells [8,19,20,21]. Par-4, which is intrinsic to normal cells, is often diminished or absent in neoplastic cells, a strategic evasion from apoptotic fate [22,23]. Suppression of Par-4 is linked to a poorer prognosis and resistance to treatment across a variety of solid tumors, underscoring its therapeutic target potential [20,24]. For instance, decreased Par-4 levels have been observed in human renal cell carcinoma and are correlated with reduced survival in patients [25,26,27]. Additionally, its knockdown in breast cancer leads to enhanced proliferation and diminished chemosensitization, highlighting the need for Par-4 reinstatement strategies [28,29,30]. The discovery of Arylquin 1 offers a beacon of hope for the treatment of cancer, given its capacity to sensitize tumor cells to apoptosis by facilitating Par-4 replenishment. This is particularly noteworthy given the complex challenges surrounding gene therapy, such as plasmid or adenoviral vector transfection. Previous accolades of Arylquin 1 include promoting morphological changes and decreasing viability across various cancer cell types; however, its effects on colorectal cancer (CRC) remain unexplored, paving the way for future investigations [21]. Par-4’s significance extends beyond its intracellular activities, notably through its extracellular presence, where it interacts with the 78 kDa glucose-regulated protein (GRP78) on tumor cell membranes to induce apoptosis [12,31,32]. Arylquin 1 plays a crucial role in this extracellular mechanism by binding to vimentin, which in turn releases vimentin-bound Par-4 for secretion [33]. This newly understood pathway not only elucidates the apoptotic efficacy of Par-4 but also reinforces the therapeutic potential of Arylquin 1 in leveraging this mechanism against cancer.

Arylquin 1 represents a promising advancement in oncological therapeutics and offers a novel approach to counteract the pervasive downregulation of Par-4 in cancer cells. Its ability to restore Par-4 levels and promote apoptosis highlights its potential as a cornerstone of cancer treatment strategies and merits further investigation to unlock its full therapeutic potential. This study of the cytotoxic effects of Arylquin 1 yielded significant insights into its therapeutic potential for the treatment of GBM. Through various in vitro and in vivo approaches, it is evident that Arylquin 1 induces a dose-dependent reduction in cell viability and proliferation in GBM8401 and A172 cells, as demonstrated by MTT and flow cytometry assays. At higher concentrations, particularly 5 μM, Arylquin 1 not only reduced viability but also increased apoptotic rates, indicating its strong antitumoral properties. In addition, Arylquin 1 was found to have a profound inhibitory effect on the invasive and migratory capacities of GBM cells. This was exemplified by the significant reduction in invasion rates and wound closure percentages of both GBM8401 and A172 cells treated with the compound compared to the untreated controls. Moreover, when combined with radiation therapy, Arylquin 1 enhanced the radiosensitivity of GBM cells, as illustrated by a significant decrease in the surviving fraction of cells upon treatment. The effect of combination therapy was most notable in in vivo models, where intracranial tumor growth was considerably suppressed, as shown by the reduced luminescence intensity in subjects receiving both Arylquin 1 and RT.

However, a limitation of this study is that, while it provides a valuable look at the cellular impact of Arylquin 1, it did not fully simulate the complex interactions within the tumor microenvironment in the body. Furthermore, the research delineates the impact of the drug at specific concentrations, but has not yet determined the optimal dosage for humans, which avoids toxicity while maintaining efficacy. Another concern is the drug delivery method, which can significantly affect bioavailability and effectiveness in the clinical context. Moreover, the timeframe of the investigation, which was extended to only 21 days after tumor implantation, did not capture potential long-term effects or delayed toxicities, leaving a gap in safety and efficacy assessments for prolonged treatment periods. Through Arylquin 1 has been demonstrated to effectively decrease the viability of cancerous glial cells, it is also apparent that it does not exclusively target cancer cells, as it impacts non-cancerous glial cells as well. This lack of selectivity necessitates the development of a more targeted approach to therapy. One promising strategy is the creation of an antibody–drug conjugate (ADC), where Arylquin 1 is linked to an antibody that specifically recognizes and binds to markers unique to glioma cells. This would endow Arylquin 1 with the specificity to target glioma cells while sparing normal cells, potentially reducing side effects and improving therapeutic outcomes. In addition to enhancing selectivity, another major challenge that needs to be addressed is the drug’s ability to cross the blood–brain barrier (BBB). The BBB is a formidable obstacle in neuro-oncology, preventing many therapeutic agents from reaching the brain at therapeutic concentrations. Arylquin 1’s limited capability to penetrate the BBB significantly hinders its clinical application. Strategies to improve its BBB permeability, or the employment of delivery systems such as nanoparticles or carrier-mediated transport, could be vital to making Arylquin 1 a viable option for clinical use in the treatment of glioma. Overcoming these obstacles is essential before Arylquin 1 can be considered suitable for clinical application.

## 5. Conclusions

Arylquin 1 is a promising candidate for improving the outcomes of GBM treatment. Its ability to enhance radiosensitivity, along with its inhibitory effects on GBM cell viability, invasion, and migration, could potentially be utilized in clinical settings to provide a more effective and synergistic approach to GBM therapy. This evidence strongly supports further exploration and development of Arylquin 1-based treatments.

## Data Availability

Data are contained within the article.
